# The Link Between the Mitochondrial Fatty Acid Oxidation Derangement and Kidney Injury

**DOI:** 10.3389/fphys.2020.00794

**Published:** 2020-07-09

**Authors:** Lara Console, Mariafrancesca Scalise, Nicola Giangregorio, Annamaria Tonazzi, Maria Barile, Cesare Indiveri

**Affiliations:** ^1^Unit of Biochemistry and Molecular Biotechnology, Department DiBEST (Biologia, Ecologia, Scienze della Terra), University of Calabria, Rende, Italy; ^2^CNR Institute of Biomembranes, Bioenergetics and Molecular Biotechnologies (IBIOM), Bari, Italy; ^3^Department of Biosciences, Biotechnology and Biopharmaceutics, University of Bari Aldo Moro, Bari, Italy

**Keywords:** mitochondria, β-oxidation, carnitine, kidney, CPT

## Abstract

Renal proximal tubular cells are high energy-demanding cells mainly relying on fatty acid oxidation. In stress conditions, such as transient hypoxia, fatty acid oxidation (FAO) decreases and carbohydrate catabolism fails to compensate for the energy demand. In this scenario, the surviving tubular cells exhibit the peculiar phenotype associated with fibrosis that is the histological manifestation of a process culminating in chronic and end-stage kidney disease. Genome-wide transcriptome analysis revealed that, together with inflammation, FAO is the top dysregulated pathway in kidney diseases with a decreased expression of key FAO enzymes and regulators. Another evidence that links the derangement of FAO to fibrosis is the progressive decrease of the expression of peroxisome proliferator-activated receptor α (PPARα) in aged people, that triggers the age-associated renal fibrosis. To allow FAO completion, a coordinate network of enzymes and transport proteins is required. Indeed, the mitochondrial inner membrane is impermeable to fatty acyl-CoAs and a specialized system, well known as carnitine shuttle, is needed for translocating fatty acids moieties, conjugated with carnitine, into mitochondrial matrix for the β-oxidation. The first component of this system is the carnitine palmitoyltransferase 1 (CPT1) responsible for transfer acyl moieties to carnitine. Several studies indicated that the stimulation of CPT1 activity and expression has a protective effect against renal fibrosis. Therefore, the network of enzymes and transporters linked to FAO may represent potential pharmacological targets deserving further attention in the development of new drugs to attenuate renal dysfunction.

## Introduction

The kidney is one of the most energy-demanding organs in the human body, as demonstrated by Elia (Elia, 1992) in a study performed in 1992 describing the specific resting metabolic rates of major organs and tissues in adults. The following values expressed as in kcal kg^–1^ d^–1^ were indeed calculated: 200 for liver, 240 for brain, 440 for heart and kidneys, 13 for skeletal muscle, 4.5 for adipose tissue, and 12 for the other organs (Wang et al., 2010). Accordingly, the kidney is second only to the heart in terms of mitochondrial abundance (Pagliarini et al., 2008) and oxygen consumption under resting conditions (O’Connor, 2006). This scenario correlates well with the high request of ATP by the kidney to remove waste from the blood, to reabsorb nutrients, to modulate the balance of electrolytes and fluid, to maintain the acid-base homeostasis, and to regulate the blood pressure (Bhargava and Schnellmann, 2017). While all kidney districts have a high request for ATP, the mechanism of ATP production is cell type-dependent. Podocytes, endothelial and mesangial cells first use glucose to produce ATP for basal cell processes (Forbes, 2016). On the contrary, proximal tubule cells, which constitute 90% of the outer kidney cortex, are the main ATP consuming cells in the kidney and use fuels such as lactate, glutamine, and fatty acids (Forbes and Thorburn, 2018). These cells are completely dependent on the oxidative phosphorylation, i.e., aerobic respiration, as the primary mechanism of ATP production (Bhargava and Schnellmann, 2017). In good agreement with such a metabolic requirement, non-esterified fatty acids, such as palmitate, are among the preferred substrates for ATP production via β-oxidation. Indeed, the efficiency of ATP production from palmitate is higher than that from glucose. Proximal tubules use the majority of the generated ATP to perform active transport and reabsorption of solutes including glucose, amino acids, phosphate, bicarbonate and various filtered low molecular weight proteins, as well as to produce the key urinary buffer ammonium (NH_4_^+^). Indeed, all the secondary active transport processes depend on the Na^+^-K^+^-ATPase localized on the basolateral side of the proximal tubular cell (Bhargava and Schnellmann, 2017). Another reason to avoid glycolysis for producing ATP is that proximal tubules harbor a high glucose concentration gradient from their luminal (urinary filtrate) to basal (blood) side for glucose reabsorption. Hence, using glucose to derive energy could be toxic, especially under conditions of metabolic imbalance such as in diabetes (Forbes, 2016). Although β-oxidation of fatty acids is the most efficient mechanism for producing ATP, this pathway implies a high oxygen request; this makes proximal tubules more susceptible than other cell types to changes in oxygen levels. Indeed, a lower oxygen supply can lead to impaired β-oxidation and ATP synthesis which in turn can trigger kidney injury. In this minireview, the network of transporters and enzymes responsible for the mitochondrial fatty acid oxidation will be dealt with focusing on derangements that underlie kidney injury with special reference to the Acute Kidney Disease (AKI).

## The Mitochondrial Fatty Acid β-Oxidation and the Carnitine Shuttle for ATP Production

**FIGURE 1:**
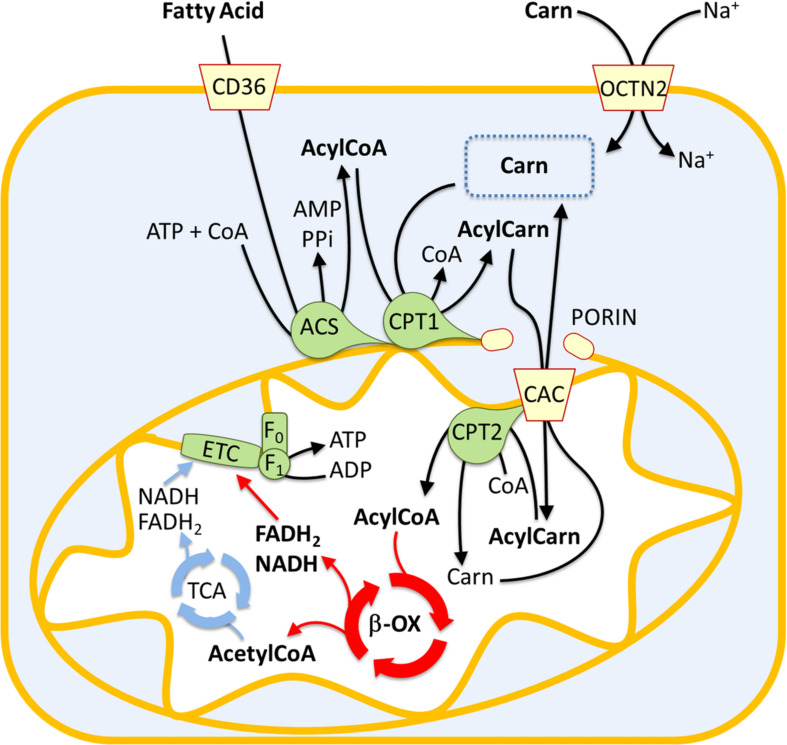
Involvement of the carnitine shuttle in the mitochondrial β-oxidation pathway. The picture represents a sketch of renal proximal tubule cells. Fatty acids (FA) enter the cytosol via CD36. FA are converted in acyl-CoA by acetyl-CoA synthetase (ACS) and then transferred to the mitochondrial matrix by the carnitine shuttle constituted by carnitine-palmitoyltransferase 1 (CPT1), carnitine-acylcarnitine carrier (CAC), and carnitine-palmitoyltransferase 2 (CPT2). Acyl-CoA undergoes β-oxidation (β-ox) with the production of acetyl-CoA that enters the tricarboxylic acid cycle (TCA). NADH and FADH_2_ generated by β-oxidation and TCA are the electron donors for the electron transport chain (ETC). The Organic Cation Transporter Novel 2 (OCTN2) mediates carnitine entry in the cytosol by a sodium dependent transport reaction.

Fatty acid oxidation mainly occurs in mitochondria and involves a repeated sequence of reactions that result in the conversion of fatty acids to acetyl-CoA. Fatty acids are mainly taken up by proximal tubule cells through CD36. This is a fatty acid transporter that plays several roles in human lipid metabolism such as fatty acids storage in adipose tissues and fatty acids supply to produce ATP by β-oxidation in proximal tubule cells (Yokoi and Yanagita, 2016; Figure 1). Alternatively, fatty acids result from the deacylation of cellular phospholipids mediated by phospholipase A2 (PLA2). Independently from their origin, fatty acids are then activated in the cytosol by the long-chain acyl-CoA synthetase producing long-chain acyl-CoA. Due to the lack of an acyl-CoA transporter in the mitochondrial inner membrane, the acyl group is transferred to the shuttle molecule carnitine for translocation into the matrix (Indiveri et al., 2011; Casals et al., 2016). The shuttling process starts with the action of CPT1 that catalyzes the conversion of acyl-CoAs into acylcarnitines (Figure 1). CPT1 was identified as part of a large protein complex of the mitochondrial outer membrane, protruding toward the cytosol, which includes long-chain acyl-CoA synthetase (ACSL) and the voltage-dependent anion channel (VDAC) also known as porin (Lee et al., 2011). The formed acylcarnitines cross the inner mitochondrial membrane through the action of the carnitine acylcarnitine carrier (CAC – SLC25A20). This transporter mediates an antiport reaction coupling the entry of acylcarnitine into the matrix with the exit of free carnitine (Figure 1). It exhibits a higher affinity for acylcarnitines with longer carbon chains and is located in the inner mitochondrial membrane where takes contact with CPT2 located on the matrix side of the inner mitochondrial membrane (Lee et al., 2011). CPT2 transfers back the acyl groups from acylcarnitines to the mitochondrial CoA forming the mitochondrial acyl-CoAs that undergo β-oxidation (Figure 1). Each round of β-oxidation requires the sequential action of at least 4 enzymes: the first enzyme is the acyl-CoA dehydrogenase (ACAD) that catalyzes the α,β-dehydrogenation of acyl-CoA. Five types of ACADs are known which are classified according to the substrate specificity; hence, each isoform has a preference for substrates with different chain lengths. These redox enzymes use FAD as a cofactor. The protein expression of medium Acyl-CoA dehydrogenase (MCAD) in the renal tubule is higher than in glomeruli, confirming the important role of FAO in this tissue (Uhlen et al., 2015). The electrons from FADH_2_ are subsequently transferred to the electron transferring factor (ETF), which in turn releases the electrons to the ETF dehydrogenase coupled with the electron transport chain via the Coenzyme Q. The second step of the β-oxidation is catalyzed by the 2-enoyl-CoA hydratase (ECH), while the third step is catalyzed by 2 distinct forms of the NAD^+^ dependent 3-hydroxyacyl-CoA dehydrogenase that show different specificities: the long-chain hydroxyacyl-CoA dehydrogenase (LCHAD) and the short/medium-chain hydroxyacyl-CoA dehydrogenase (HAD). The fourth step is catalyzed by the long-chain 3-ketoacyl-CoA thiolase (HADHB) with a broad chain-length preference. After this first round of reactions, the two carbon shortened Acyl-CoA is recycled through subsequent rounds of β-oxidation reactions generating Acetyl-CoA that enters the TCA cycle allowing the ATP synthesis by the oxidative phosphorylation (Figure 1). As above described, the availability of carnitine for fatty acid transport into mitochondria is crucial for the accomplishment of FAO. Due to its fundamental role, it is not surprising that the carnitine homeostasis is strictly regulated (Pochini et al., 2019). The endogenous carnitine synthesis is insufficient to meet the human body need, thus, about half of the carnitine pool is absorbed from the diet. The plasma membrane Organic Cation Transporter Novel 2 (OCTN2 – SLC22A5), is the major player for carnitine absorption and distribution to the various tissues. The transporter also plays a major role in the renal tubular reabsorption of carnitine which concurs in maintaining the homeostasis (Pochini et al., 2019). Kidney diseases are associated to some extent with carnitine homeostasis derangements. Indeed, some patients with kidney diseases develop carnitine deficiency (Hedayati, 2006).

## Fatty Acid Oxidation Impairment and the Onset of Kidney Diseases

**FIGURE 2:**
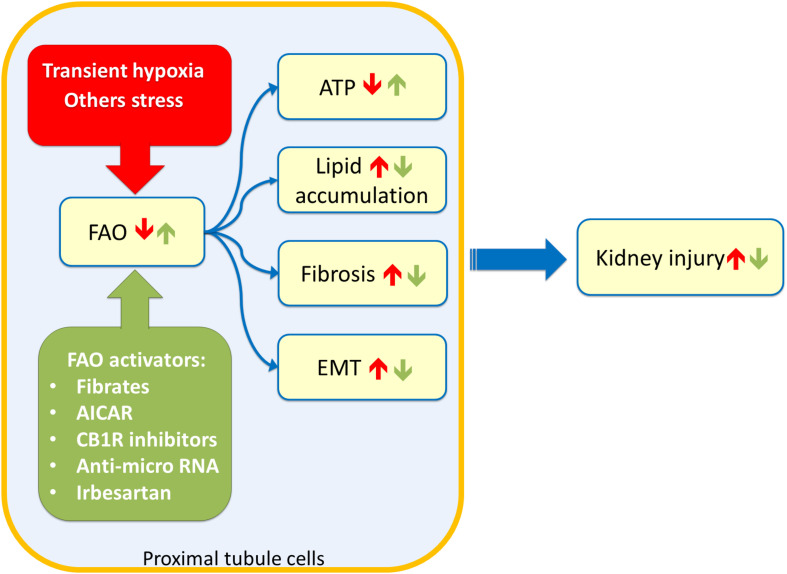
Sketch of kidney tubule cell injury. Stress such as transient hypoxia (red box) can impair Fatty Acid Oxidation (FAO) of proximal tubule cells leading to ATP depletion, lipid accumulation, fibrosis phenotype, and Epithelial to Mesenchimal Transitiona (EMT). These events culminate in chronic as well as acute kidney injury. Stimulation of FAO trough drugs or innovative therapy approaches (green box) can ameliorate the kidney functions after insults. 5-aminoimidazole-4-carboxyamide ribonucleoside (AICAR); renal cannabinoid-1 receptor (CB1R).

The link between lipid accumulation and kidney disease was suggested for the first time in 1860 by Rudolf Virchow. In a lecture at the Pathological Institute of Berlin, he mentioned the “fatty degeneration of the renal epithelium as a stage of Bright’s disease,” which is the historical name for glomerulonephritis indicating a range of immune-mediated disorders that cause inflammation of glomeruli and other compartments of the kidney (Chadban and Atkins, 2005). Lipid accumulation reflects an imbalance between fatty acid utilization and fatty acid supply. Since triglycerides buffer fatty acid excess, the overload is often visible as lipid droplets. Several studies suggested that glomeruli and proximal tubules are the most susceptible to lipid accumulation and hence linked to kidney dysfunction (Figure 2; Bobulescu, 2010). A typical example is the diabetic nephropathy (DN), a life-threatening pathological condition occurring in the 50% of diabetes mellitus patients with End Stage Renal Disease (ESRD). The molecular features of DN are not yet completely understood; however, lipotoxicity caused by fatty acid deposition and tubule-interstitial fibrosis characterized by epithelial-to-mesenchymal transition (EMT), seem to be hallmarks of DN. Interestingly, a study conducted on human proximal tubular cells, cultured in high glucose medium to mimic diabetic condition, shed light on the events underlying DN onset. It seems that the lipotoxicity occurs before the EMT (Xu et al., 2014). In line with this, the silencing of a key enzyme for fatty acid biosynthesis, namely acetyl-CoA carboxylase 2 (ACC2), increased the β-oxidation rate with a reduction of lipotoxicity and reversion of the EMT morphological changes (Xu et al., 2014). The effect on the β-oxidation rescue is due to reduced production of malonyl-CoA by ACC2 with consequent activation of CPT1, un upstream enzyme of FAO (Figure 1). In line with this, another study reported that tubular epithelial cells treated with the CPT1 inhibitor etomoxir showed an increase of gene expression typical of fibrosis, such as α-SMA and Vimentin (Kang et al., 2015); conversely, a model of induced kidney fibrosis treated with the CPT1 activator C75 showed an improvement of the fibrosis phenotype (Kang et al., 2015). Another pathological condition characterized by fatty acid accumulation causing lipotoxicity is one of the most common forms of acute kidney injury (AKI), which is the ischemic renal injury (IRI). The mechanism of lipid accumulation in IRI is not completely understood. Indeed, the accumulation of triglycerides and cholesterol, following the onset of ischemia, seems protective due to the buffer effects against fatty acids. Later, with the progress of the ischemia, the lipid accumulation, visible as droplets, may cause acute kidney injury (Erpicum et al., 2018). During ischemia/reperfusion injury, a decline in the activity of CPT1 has been observed resulting in reduced uptake of fatty acids in the mitochondrial matrix and, hence, reduced FAO. Treating kidney with the CPT1 activator C75, the FAO increased with consequent improvement of renal morphology (Idrovo et al., 2012b). The IRI is characterized by an increase in oxidative stress. This has an influence also on the activity of the central component of the carnitine shuttle, CAC (Figure 1). This membrane transporter is located in the inner mitochondrial membrane and possesses six cysteine residues two of which, namely C155 and C136, constitute a redox sensing centre. Indeed, H_2_O_2_ at toxic concentrations, triggers the formation of a disulfide bridge between C155 and C136 switching off the transport activity. In this respect, it is interesting that the gas transmitters NO or H_2_S as well as GSSG act on the same cysteines modulating the CAC activity. This, in turn, may influence the mitochondrial uptake of fatty acids (Giangregorio et al., 2013, 2016; Tonazzi et al., 2013, 2017). In line with this, preconditioning with propionyl-carnitine, to replenish the carnitine pool for FAO, is protective against IRI damage in a rat model (Erpicum et al., 2018). Another event occurring during IRI and, then, characteristic of AKI pathogenesis, is hypoxia (Figure 2). In this condition, the NADH: NAD^+^ ratio in the mitochondria increases due to the reduction in NADH oxidation rate in the respiratory chain (Simon and Hertig, 2015). Indeed, in the absence of adequate oxygen supply, the electron transport chain is slowed down with the accumulation of NADH which, in turn, inhibits NADH-producing reactions. These phenomena have important impacts on lipid metabolism: for example, the above-mentioned 3-hydroxyacyl-CoA dehydrogenase is one of the rate-controlling enzymes of FAO and requires NAD^+^ as a cofactor; this enzyme is impaired when the NADH: NAD^+^ ratio increases with the reduction of FAO and lipid accumulation (Simon and Hertig, 2015). In good agreement with the existence of a coordinate network between enzymes and transporters for accomplishing FAO (Figure 1), the expression of these proteins is mainly under the control of the same group of transcription factors, namely peroxisome proliferator-activated receptors PPAR-α, PPAR-β/δ, and PPAR-γ. The PPARs are predominantly expressed in metabolically active cells, such as the renal proximal tubules (Varga et al., 2011). PPARs can recognize a large class of endogenous ligands among which some ω3 and ω6-polyunsaturated fatty acids (PUFAs) and some saturated fatty acids, such as myristic acids. PPARs interact with the members of another subfamily of nuclear receptors, namely RXRs (retinoid X receptor), forming heterodimers. Upon this binding, the heterodimer translocates in the nucleus and interacts with specific DNA responsive elements with consequent increased expression of FAO enzymes as well as of the fatty acid transporter CD36. The plasma membrane transporter OCTN2 and the mitochondrial transporter CAC are also regulated by some of the PPAR family members, among which PPAR-α (D’Argenio et al., 2010; Indiveri et al., 2011; Varga et al., 2011; Qu et al., 2014; Zhou et al., 2014). Interestingly, a link between lipid accumulation, AKI, and PPARs has been demonstrated. Indeed, the renal fibrosis and the epithelial-to-mesenchymal transition, characteristic of AKI are induced by the secretion of the transforming growth factor β1 (TGF-β1) from tubular epithelial cells. TGF-β1 is a cytokine that reduces the expression of PPAR-α and the PPAR-γ coactivator-1a (PPARGC1A) with consequently reduced expression of CPT1 and of the FAO enzyme Peroxisomal acyl-coenzyme A oxidase 1 (Acox1) which catalyzes the desaturation of acyl-CoAs to 2-*trans-*enoyl-CoAs. The effects of TGF-β1 are exerted through the transcription factor SMAD3 which binds to an intronic region of PPAR-α gene. This interaction affects the expression of all the downstream targets causing significant metabolic depression, low cellular ATP content and, then, lipid accumulation (Kang et al., 2015). It has also been demonstrated the microRNA-21, upregulated in different models of renal fibrosis, can silence PPAR-α expression triggering FAO blockade and lipotoxicity. Accordingly, miR-21(−/−) mice suffered less interstitial fibrosis in response to kidney injury (Chau et al., 2012). Therefore, miR-21 and PPAR-α act coordinately in regulating FAO in renal injury. Another microRNA, miR-33, seems to have a role in lipid accumulation and development of fibrosis in the kidney (Price et al., 2019).

## Therapeutic Interventions for Rescuing Fatty Acid Oxidation

As described above, dysfunctions of the mitochondrial β-oxidation trigger kidney injury, therefore, mitochondria represent a potential drug target. Several novel compounds that promote mitochondrial function are currently under development (Szeto, 2017). A growing number of data suggests that the activation of PPARα can be protective (Cheng et al., 2010). The first class of compounds tested to obtain PPARα activation was that of fibrates (Figure 2). In a murine model, it was demonstrated that the treatment with high-dose of PPARα agonists worsened the tubular damage; while, pre-treatment with a low dose of clofibrate prevented acute tubular injuries without accumulation of metabolites harmful for renal function. The tubular protective effects appeared to be associated with the counteraction of PPARα deterioration, resulting in a maintenance of FAO, a decrease of intracellular accumulation of undigested FFAs, and attenuation of disease developmental factors including oxidative stress, apoptosis, and NFκB activation (Takahashi et al., 2011). These effects are common to other fibrates, such as fenofibrate, that significantly attenuated the degree of renal dysfunction and inflammation caused by ischemia/reperfusion (I/R) injury. Interestingly, fenofibrate did not protect PPAR-α(−/−) mice against I/R (Patel et al., 2009). Furthermore, the PPAR- α agonist, WY14643, seems to reduce the renal dysfunction and injury associated with I/R (Sivarajah et al., 2002), while benzafibrate inhibits cisplatin-mediated injury by preventing the proximal tubule cell death (Nagothu et al., 2005). An alternative to fibrates is represented by the angiotensin II receptor blocker irbesartan, which increased PPARα signaling in the liver as well as in the kidney (Figure 2). The effect of irbesartan seems to be safe also for the patient with CKD (Harada et al., 2016). Peroxisome proliferator-activated receptor γ coactivator 1α (PGC-1α) is another key transcriptional regulator of mitochondrial biogenesis and function (Li and Susztak, 2018). A decrease in PGC-1α expression is commonly observed in mice and patients with acute and chronic kidney disease. Increasing PGC-1α expression in renal tubule cells restores energy deficit and has been shown to protect from acute and chronic kidney disease. Stimulation of the PGC-1α pathway could provide a potential intervention strategy, however, endothelial cells and podocytes are negatively influenced by excessive increased PGC-1α (Li and Susztak, 2018). Another approach to improve renal function after IRI is represented by a treatment combining carnitine and 5-aminoimidazole-4-carboxyamide ribonucleoside (AICAR), which activates CPT1 through the adenosine monophosphate-activated protein kinase (AMPK). This combined treatment significantly increased CPT1 activity and ATP levels with lowered renal malondialdehyde, a marker of oxidative stress, and serum TNF-α levels (Idrovo et al., 2012a). An additional strategy for rescuing the ATP level after AKI is the treatment with the mitochondria-targeted compound SS-31; it belongs to the Szeto-Schiller (SS) peptides family, discovered by Hazel H. Szeto and Peter W. Schiller. SS-31 selectively targets and concentrates in the inner mitochondrial membrane where binds with a high affinity the anionic phospholipid cardiolipin (Szeto and Schiller, 2011). Interestingly, cardiolipin plays a structural role in the cristae formation, in the organization of the respiratory supercomplexes and is essential for the transport activity of CAC (Tonazzi et al., 2005). The SS-31/cardiolipin complex inhibited peroxidase activity of cytochrome c, which catalyzes cardiolipin peroxidation and results in mitochondrial damage, FAO decrease and lipid accumulation (Birk et al., 2013; Szeto, 2014, 2017). A novel class of therapeutics is represented by the anti-miR oligonucleotides (Figure 2); indeed, several advancements have been made in RNA stability *in vivo*. Interestingly, the latest generation of anti-miR oligonucleotides can be delivered by weekly subcutaneous injection without loss of activity. Recent studies using anti-miR-21 and anti-miR-33 oligonucleotides demonstrated protective renal action (Chau et al., 2012; Price et al., 2019). In particular treatment with anti-miR-33 significantly increases protein levels of CPT1a (the kidney and live isoform) as well as Peroxisomal carnitine O-octanoyl transferase (CROT) which are involved in FAO, reducing the development of fibrosis (Price et al., 2019). A great deal of attention is currently placed on the effects of obesity on the development of kidney disease. Obesity-induced nephropathy is linked to the activation of the renal cannabinoid-1 receptor (CB1R). Inhibition or selective deletion of CB1R in the renal proximal tubule cells markedly attenuated lipid accumulation, inflammation, and fibrosis in the kidney (Figure 2). These effects are associated with enhanced fatty acid β-oxidation and increased activation of the liver kinase B1/AMP-activated protein kinase signaling pathway (Udi et al., 2017).

## Conclusion

Mitochondrial dysfunction is commonly observed in many nephropathies. Renal cell repair is dependent on the ability of mitochondria to rescue the production of ATP. Thus, restoring mitochondrial β-oxidation might reverse or attenuate renal failure. PPAR-α agonists prevent tubule cell death and intracellular lipid accumulation. In this scenario, a deeper understanding of the tissue specificity effects and the molecular mechanisms of PPAR-α is mandatory to develop novel and safer therapies. The most innovative pharmacological approach is the use of anti-micro RNA oligonucleotides. This new strategy can now be suitable thanks to the advancement in the stability of these molecules. A weekly subcutaneous injection is sufficient to deliver the anti-miR without loss of activity.

## Author Contributions

LC contributed to collecting bibliography, writing the manuscript, and conceiving and creating the figures. MS contributed to writing the manuscript and creating the figures. NG, AT, and MB were involved in the critical revision and writing of the manuscript. CI supervised the work and wrote and revised the manuscript. All authors contributed to the article and approved the submitted version.

## Conflict of Interest

The authors declare that the research was conducted in the absence of any commercial or financial relationships that could be construed as a potential conflict of interest.
